# The contribution of antimicrobials and antimicrobial resistance to climate change and a possible way to reverse it whilst still offering high quality healthcare—a conceptual analysis

**DOI:** 10.3389/fpubh.2025.1644086

**Published:** 2025-07-15

**Authors:** Jeanette Sams-Dodd, Frank Sams-Dodd

**Affiliations:** Willingsford Ltd., Southampton, United Kingdom

**Keywords:** climate change, antimicrobial resistance, antibiotics, One Health, MPPT, mobile genetic elements, greenhouse gases, infection prevention and control

## Abstract

Since 1954, studies have consistently demonstrated that antimicrobials disrupt microbial environments, causing ecosystem degradation and release of greenhouse gases (GHG), making antimicrobials noteworthy climate stressors. Microbes created an atmosphere on Earth that supports eukaryotic life-forms and are essential for our normal physiological functions. However, despite their critical importance, microbes are mostly associated with infectious diseases, and antimicrobials are extensively used to eradicate them. In healthcare and veterinary medicine, antimicrobials are essential in fighting infections. The general risk associated with their use has focused on antimicrobial resistance and loss of efficacy, whereas their impact on microbial environments and GHGs has been overlooked. Using recent data, a single course of antibiotics is estimated to cause the release of 9.84 tonnes of CO_2_—the equivalent of a standard car driving around the Earth 1.47 times. Given the number of chemicals with antimicrobial effects, such an amount demands attention. Antibiotics, antiseptics, disinfectants, surfactants as well as pesticides, herbicides and many food additives all contribute to antimicrobial-resistance. Despite a focus on antibiotic stewardship, antimicrobials are still used indiscriminately, including where they fail to confer a critical or even demonstrable benefit. Using a One-Health approach, this manuscript provides a non-specialist introduction to the microbial environment and the impact of antimicrobials, and suggests how to minimise the environmental impact of healthcare whilst retaining quality care. Climate change is assumed to contribute to AMR, but this analysis finds that AMR strongly contributes to climate change, i.e., the reverse of the normal assumption. The current climate debate almost exclusively focuses on fossil fuel without in earnest considering other sources. However, without including the major, natural systems that significantly impact the climate, balanced informed decisions to mitigate the situation are impossible to make. By forcing the focus of the climate discussion onto only a narrow, limited set of explanations, the proposed solutions will likely not solve the main causes and their impact is therefore bound to be minimal. This is comparable to symptomatic versus curative treatment in healthcare. Whereas symptomatic treatment can help alleviate, it does not address the root cause and, therefore, cannot restore the patient to health.

## Introduction

*“We live in a microbial driven world that only exists because Bacteria and Archaea tempered the previously hostile environment on early Earth to create atmospheric conditions that allow eukaryotic life forms to flourish. Bacterial and archaeal encoded enzymes catalyze all the major processes involved in global biogeochemical cycling, playing key roles in the carbon and nitrogen cycles, and producing approximately half of the oxygen in the Earth’s atmosphere.”* Clokie et al. (1).

The Earth’s environment is primarily the result of the action of microbes. Microbes are responsible for creating an atmosphere that can sustain eukaryotic life forms such as mammals (1–3). Higher life forms also directly depend on microbes, e.g., microbes in the gut provide essential compounds for our health, and, to protect us, the immune system and microbes interact directly and symbiotically (4–6). Over time however, the image of microbes has been shaped mainly by the fact that they can cause infectious diseases with the result that our goal has become the general eradication of microbes. This has resulted in the development of an array of synthetic microbe-killing compounds, i.e., antimicrobials, such as antibiotics, antiseptics, antifungals, antivirals, antiparasitics, disinfectants, surfactants, pesticides, and herbicides, all of which are used rather indiscriminately. However, when a group of organisms is exposed to a sustained selection pressure, they develop strategies to counteract this pressure, in microbes this would, for example, be antimicrobial resistance (AMR) (7). However, this change can also affect the functions these organisms were performing, thereby initiating large-scale and far-reaching change. All antimicrobials cause AMR, including cross resistance between types of antimicrobials, e.g., an antiseptic results in AMR to itself and other antiseptics as well as to antibiotics and vice versa (8); and antivirals also contribute to bacterial resistance (9). The use of any antimicrobial can, therefore, lead to broad, widespread resistance and unpredicted functional changes at a global level.

In humans, the use of antimicrobials has been found to have wide ranging negative consequences for our health. For example, a single course of antibiotics has been found to alter the gut microbiome with these changes still being detectable after 2 years in adults and permanent in infants (10). These effects can give rise to long-term health issues in the individual such as an increased prevalence of cancer, obesity, diabetes, asthma, and mental health issues. However, they also affect the next generation by increases in the prevalence of miscarriages, foetal malformations, and in children they can cause functional impairments in their development, immune function, and cognition, including attention deficit hyperactivity disorder (ADHD) (10–13). Many food additives also possess antimicrobial properties and can affect the gut microbiome, e.g., monosodium glutamate (MSG) is antimicrobial (14, 15) and is also used in animal models to mimic childhood obesity (16–18).

A general characteristic of human-manufactured antimicrobials is that they are chemically highly stable. Antiseptics and surfactants are not metabolised but enter nature unchanged, and most antibiotics, that are consumed and excreted, are essentially excreted either unmodified or as active metabolites in wastewater. As sewage plants only remove a fraction of these chemicals, the result is that they spread via the waterways and impact the microbial environments across the Earth. As early as 1954, it was shown that the addition of antibiotics (19) to soil releases substantial amounts of CO_2_ and calculations using recent data (20) indicate that antibiotics may have reduced long-term CO_2_ storage capacity by 194.8 billion tonnes in fertile land areas alone, meaning that 7.3% of the CO_2_ currently present in the atmosphere would, without the dissemination of human-made antimicrobials, still be stored in fertile land areas. The number is equivalent to 5.2 times the total calculated CO_2_ emissions from human activity in 2022 (21).

Antimicrobials are important in healthcare but they are frequently used without regard to the fact that they will cause a much wider impact than the one on the infection that they are sought to treat. One Health is based on the intertwined, interdependent relationship between humans, animals, plants, and the environment, the recognition of which is a requirement for the wellbeing of all. The aim of this analysis is to focus on the environmental aspects of antimicrobials used in the pursuit of human and animal health, potentially at the expense of the environment. Particular attention will be brought to climate change, which traditionally has been attributed mainly to fossil fuel, arguing that our use of antimicrobials may play a very significant role in the changes to the climate, that we see.

First, a brief introduction to microbial communities, antimicrobials, antimicrobial resistance, the spread of antimicrobials and their environmental implications will be given. Next, it will focus on the effect of antimicrobials on greenhouse gases (GHGs) and carbon storage. This is followed by a brief description of the spread of antimicrobial resistance and the impact of antimicrobials on individual microbial populations, including their recovery. Finally, strategies to reduce our impact on the environment, whilst still offering effective healthcare, will be discussed. The importance of choice of treatment approach in healthcare in relation to climate change is a factor that rarely is raised, but data show that it may play a much greater role than we have realised previously.

## Microbiomes, antimicrobials, and their impact

### Microbiomes

Bacteria and other microbes are everywhere around us, including in the air, soil and water. Some live in a free-floating planktonic form, but most settle and group into communities called microbiomes, which, among others, include bacteria, archaea, fungi, protists, viruses and, in aquatic systems, algae. In these ecosystems, they live in synergy, dividing tasks and depending on each other (22–28). Everything on Earth has its own microbiome, ranging from the stone on a beach to the leaf on a plant and to the cloud (24, 29–31).

The composition of each microbiome depends upon what it lives on and in. It changes constantly according to changes in its micro-environment, e.g., temperature, humidity, sources of nutrition, acidity, hormones, light intensity, and availability of the gases on which its microbiota, i.e., the species making up the microbiome, is dependent, e.g., O_2_, H_2_, N_2_, and CO_2_ (9, 32, 33).

Living surfaces are protected by microbiomes. Microbiomes are an integral part of each living surface and keep it healthy and functional (34, 35). If the microbiota is altered too much, rapidly, or violently, the microbiome is thrown off balance, also called dysbiosis, and infection may develop, resulting in the surface deteriorating and, in severe cases, dying.

A microbiome is not limited to a population living strictly on top of the surface; they usually extend well into the underlying mass, without forming a well-defined border. An example is the human skin, where different microbes thrive at different depths (36). More radical examples are soil, oceans and lakes. Here the microbiome forms an integral part of the entire space, with the microbial population changing in accordance with the varying conditions at each depth from the surface (37). Other examples are the atmosphere and clouds, where variations in microbiome composition change with height above the ground (38).

### Antimicrobials and AMR

“Antimicrobials” is the common denominator for all chemical substances that kill microbes. In healthcare, it includes antibiotics, antiseptics, antifungals, antivirals, antiparasitics, disinfectants, surfactants and biofilm based therapies (9, 33, 39, 40). Since 1949, antibiotics have been known to affect the environment (41).

AMR occurs when microbes have, develop or acquire the ability to withstand antimicrobials at the concentration they are exposed to in their environment.

Some microbes possess the ability to withstand or tolerate certain antimicrobials, and some microbes are effective at developing tolerance to antimicrobials. Microbes can upscale these abilities very quickly, and efficiently donate and share such tolerance genes with other microbes, including between species and across host types and host environments, so that many other microbial species acquire AMR capabilities (42–45). Due to virulence factors often being located in proximity to resistance genes on the genetic elements being shared such development of increased tolerance is frequently associated with increased virulence—in effect making the microbes stronger and more invasive of their surroundings and host surface, i.e., more dangerous (46–50).

**Figure 1:**
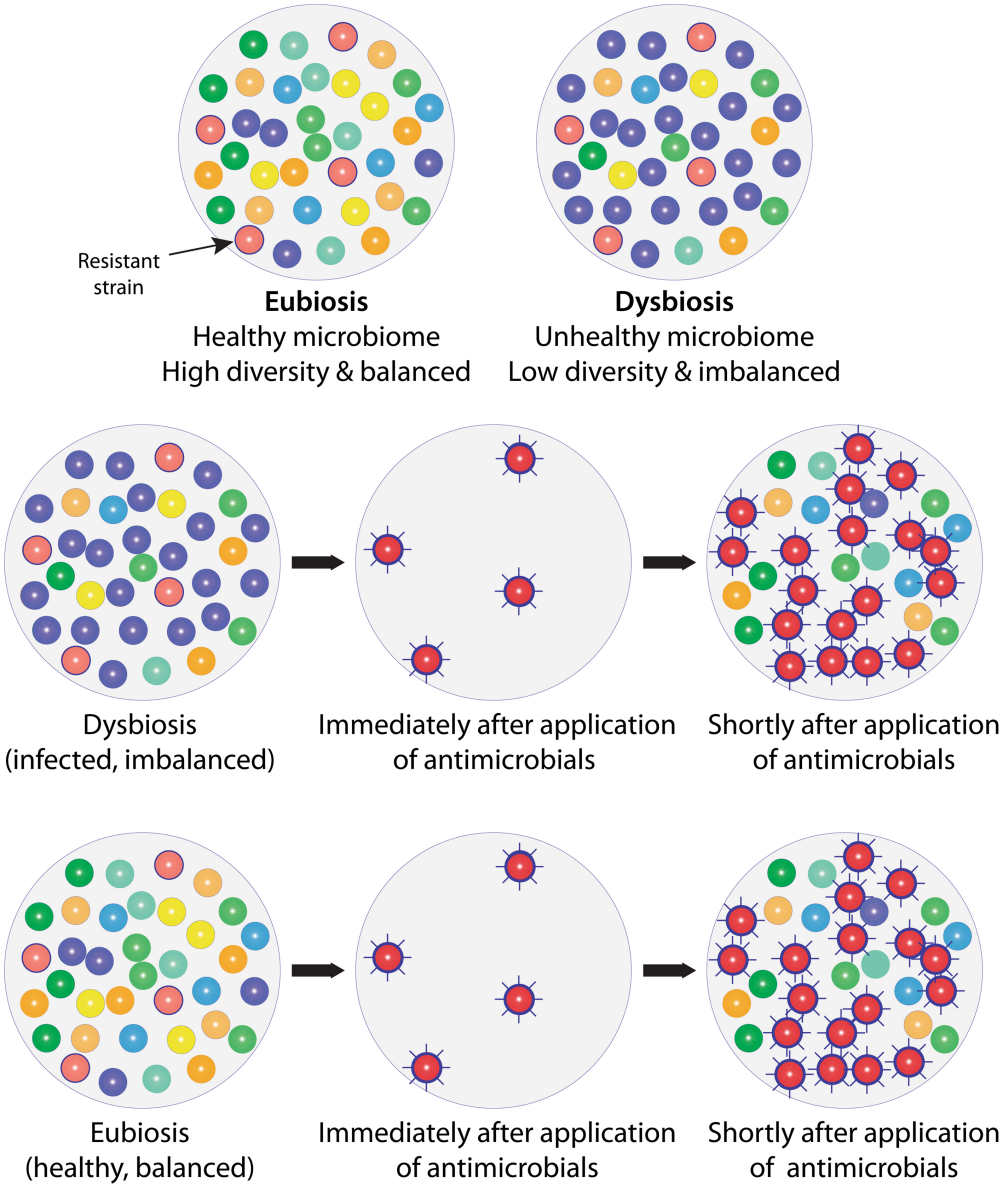
Balanced, healthy microbiomes in the state of eubiosis vs. imbalanced, unhealthy microbiomes in the state of dysbiosis, and the impact of antimicrobials. Top row: To the left is shown a natural, healthy microbiome, which is diverse and balanced, i.e., in a state of eubiosis; and to the right an imbalanced (infected) microbiome in dysbiosis, displaying low diversity and one strain (purple) dominating which would indicate that specific strain has taken over control, leading to a change in relative abundances. An antimicrobial-resistant strain is present (red with border) in both microbiomes, but this does not cause a problem as no antimicrobials are present or being administered. Middle row: The imbalanced (infected) microbiome is exposed to antimicrobials. Only the resistant strain (red with border) survives and becomes more virulent (spikes). Shortly after antimicrobial exposure is discontinued, more strains start repopulating the area, but they are at a disadvantage, making it more difficult to gain a foothold. Some species will no longer be present and others only in reduced numbers and this loss in diversity may never be restored (fully recover). Not only has the imbalance (dysbiosis) not been solved, but the resistant and more virulent strain has taken over as the dominating, imbalance-causing, i.e., infecting, strain. The microbial composition, i.e., diversity and relative abundance, has therefore shifted permanently, which, inter alia, is likely to cause a change in the overall microbial metabolism with potentially far-reaching knock-on effects. Bottom row: When providing antimicrobials, these also inadvertently reach healthy areas. The effect of the antimicrobials on healthy microbiomes will be the same as on unhealthy microbiomes, i.e., sensitive strains will be removed and resistant, frequently more virulent strains will not only remain but be provided a competitive advantage. Antimicrobials, therefore, leave previously healthy, balanced microbiomes in dysbiosis, i.e., cause instability and possible infection in hitherto healthy areas. To simplify, only bacteria are included in the example. AMX: Antimicrobial.

When an antimicrobial is used (Figure 1), only resistant species will survive and remain. This leaves the area open to expansion and provides the surviving resistant species a competitive advantage by providing the opportunity for quick expansion into this newly unoccupied territory to seek dominance. The original species of the microbiome will also seek to repopulate the area and restore health, but they will be at a disadvantage as the resistant species will have had a head start. The path back to eubiosis with a balanced (51), highly diverse (52), mutualistic (53, 54) microbial ecosystem will, therefore, take time and the original state may never be reached—the composition of the microbiota, i.e., the species making up the microbiome, may have permanently changed, causing long-term consequences (see Introduction). Finally, during this period of upset and disruption of the microbiome, it will be easier for passing potentially inherently pathogenic microbial species to gain a foothold.

### Impact of antimicrobials and AMR on food webs and biogeochemical cycles

Microbes are very diverse in their nutritional and gaseous needs, and the metabolic products that they manufacture, are also highly diverse. Whilst we often describe these as break-down products, they are usually not waste products but of great value to other microbial species (4, 55–57).

**Figure 2:**
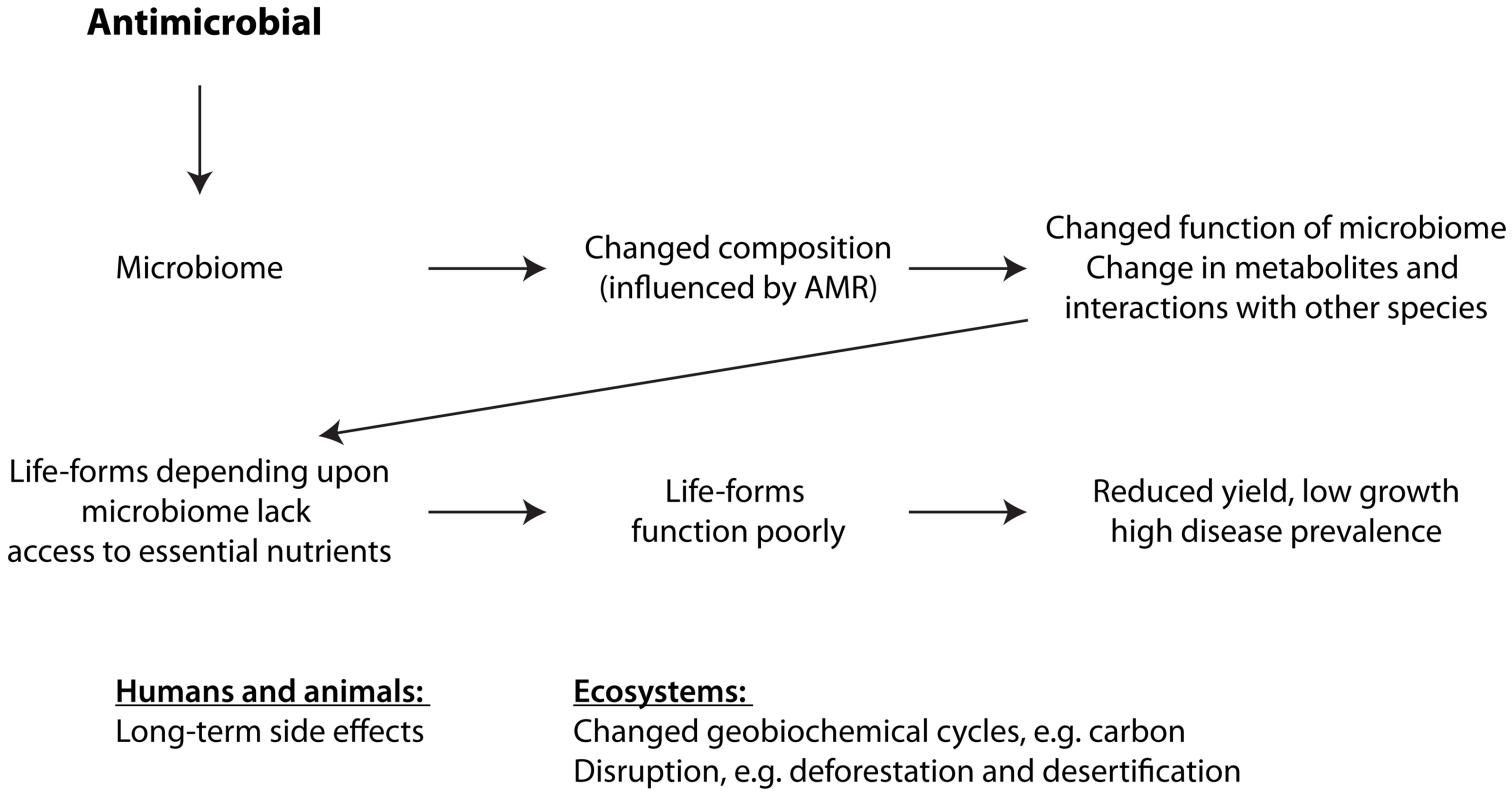
How antimicrobials affect ecosystems. See text for explanation. Changes to the composition of the microbiota, i.e., the species making up the microbiome, affect how the microbiome supports other organisms directly dependent upon it, causing these higher, dependent organisms to function suboptimally.

With the change in composition of the microbiota, the overall consumption of resources will differ in their proportion from how they were utilised previously by the original, pre-antimicrobial microbiome (58). With some species now extinct, others fewer in number, and antimicrobial resistant species now in abundance, some resources will become superfluous while other resources will be prematurely exhausted. Similarly, some metabolites may become excessive whilst others will be missing for the optimal functioning of the microbiome, e.g., changes to the composition of the root microbiome impact plants, e.g., crop yields, growth, and response to pathogens (59–62). AMR therefore leads to a severe imbalance in ecological systems that rely on a microbiome to release, absorb and retain certain gases, moisture, and particular nutrients in the appropriate amounts (Figure 2).

Microbiomes are at the very bottom, i.e., the first, foundational step on the ladder, of all food chains in all ecological systems, and they play a vital role in all the biogeochemical cycles, e.g., water, carbon and nitrogen cycles (63, 64). Antimicrobials, consequently, directly or indirectly, influence the functioning of all ecosystems and biogeochemical resource cycles through the creation of AMR.

### Spread of antimicrobials and AMR

**Figure 3:**
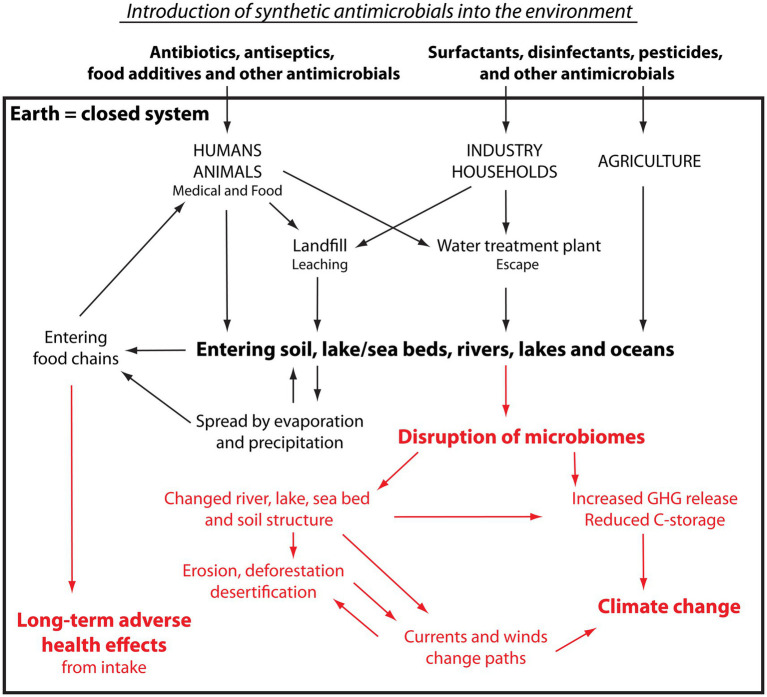
Pathway and impact of synthetic antimicrobials introduced into the environment. Please see the text for explanation. Black: Pathway of antimicrobials. Red: Impact on living organisms and ecosystems.

As illustrated in Figure 3, antibiotics and antiseptics are used in the treatment of patients in hospitals, across community care, and in veterinary care (Figure 3). Antiseptics, disinfectants, and surfactants are used for cleaning in hospitals, clinics and—after Covid—even more widely in societal communal areas such as train stations, schools, and community centres. Their use is also abundant and widespread in a large number of industries such as textiles, agrochemicals, paints, lubricants, household detergents, personal care, plastics, industrial cleaning and many more, often with no evidence of benefitting.

Generally, antibiotics are small molecules excreted intact, i.e., unmetabolised, or only partly metabolised through the urine or faeces and, similarly to antiseptics, disinfectants and surfactants, they are typically very stable chemicals having been selected for a long half-life in order to be convenient to distribute and store (65). Antimicrobials usually possess very strong binding properties, and studies have shown that the biological activity of antibiotics continue when bound to organic materials and that binding to organic matter increases their half-life (66).

Upon use, antimicrobials usually make their way to sewage plants or landfill sites. The antimicrobials on landfills and fields enter the wider environment unhindered, carried by water leaching through the soil (67). As sewage plants typically retain around 50% of the antimicrobials unmodified, all microbes that pass through the sewage plant will be exposed to high levels of many different antimicrobials, thereby substantially increasing their probability of developing AMR (68). The other half of the antimicrobials, i.e., those that are not retained, are released from the water treatment plants into the aquatic environment (58, 67, 69, 70). Having now entered the water cycle, they travel unhindered in water moving through soil, surface water, rivers, lakes and oceans as well as bind to the creatures that they pass. They are, consequently, widely and effectively dispersed (71–73). On their path, they are also taken up by plants and animals growing and living in the affected soil and water and by animals drinking from the water (69, 74), and the spread is helped even further by predators (75–78). These recycled antimicrobials are returned to humans via water and food, meat-based as well as plant-based (71, 79, 80). The antimicrobials are also carried by water evaporating from surfaces into the air, where they, by wind and clouds, are carried through the atmosphere before being released back to Earth as precipitation, including over areas uninhabited by humans (30, 81–84)—where they can now embark on a new cyclical journey. At each step of the journey, the antimicrobials will have induced AMR as well as have caused varying degrees of dysbiosis in the microbiomes they have been in contact with.

### Implications

As the microbial populations change composition (Figure 3, red text), their tasks of converting essential nutrients, e.g., nitrogen, in the adequate order and at a speed adjusted to the seasonal requirements get out of sync (56, 85–89). All food webs rely on such conversions and, as a result, soil, lake and ocean floors die away, changing their structure from a porous air-filled sponge to a lifeless compact brick in which animals cannot live, and plant roots cannot penetrate to gain a foothold. The exhausted upper soil, sea and lake floors erode causing deforestation, leaving behind deserts of non-fertile land, lake and ocean beds. This again causes a change in availability of photosynthetic organisms to capture the CO_2_ from the atmosphere and store the carbon. Therefore, crippling the microbiomes in soil and waterways causes increased release of the greenhouse gases (GHGs), including nitrous oxide (N_2_O), methane (CH_4_), and carbon dioxide (CO_2_), into the atmosphere and reduces carbon capture and storage (19, 45, 56, 90–95).

The deterioration of the ocean floor causes the main currents to change their usual paths (2, 92, 96). Apart from severely impacting the long-established marine ecosystems, this modifies the travel paths of the winds (97) thereby changing the traditional precipitation patterns. In addition, the microbial imbalance influences the geographical locations where water will be released, leading precipitation to bypass certain areas, depriving them of water and causing drought and desertification, which further impedes carbon sequestration and storage. In contrast, other areas will receive precipitation in abundance leading to flooding. Furthermore, different microbial species respond differently to the cold, and, as the antimicrobials change the microbial composition and proportions in the clouds, it changes the nature of precipitation, e.g., whether it will fall as rain, snow or hail, including the number, size and weight of the droplets and hails (98–100). This can bring about thundersnow, heavy hail, and torrential rainfall which all lead to further erosion, landslides, and deforestation, thereby releasing more stored carbon into the atmosphere (101, 102).

In essence, the synchronised collaboration of the trillion species (102) constituting the microbial population in air, soil, and water is essential to the coordinated cycling of all nutrients. The synchronisation of this plethora of tightly intertwined and interdependent processes, which have developed over an evolutionary timeframe, is critical for the nutrients to be available in the right form, at the right place, at the right time and in the right amount. In other words, the microbial communities are the main regulators of the major Earth biogeochemical cycles, including water, carbon, nitrogen, oxygen and phosphorus (102). Release of antimicrobials into the environment, therefore, significantly unbalances the microbial communities and desynchronises these cycles—and thereby change the climate.

## Earth climate, greenhouse gasses and AMR

### Earth, her atmosphere and GHGs

Our planet sits in Space and consists of the Earth with a surrounding atmosphere that extends from the Earth’s surface and into Space. It is a closed system with essentially fixed amounts of the different types of molecules, e.g., there is a fixed amount of carbon, which must be in either the atmosphere or the Earth. The baseline temperature of outer space is 2.7 kelvins, which is the equivalent of minus 270.45°C, and the temperature of the Sun’s surface is around 15 million °C. Most life forms on Earth require temperatures in the general range from 0°C to 50°C, so the acceptable temperature range on Earth is extremely narrow compared to the surrounding environment in Space. Life on Earth uses and generates gases, including carbon dioxide, methane and nitrous oxide, whose physical properties make them insulators that retain heat around the Earth, i.e., creating a greenhouse effect. They are therefore referred to as Greenhouse Gases (GHGs). During the day, sunlight heats up the atmosphere and during the night, heat is lost to Space. Because some gases are better insulators than others, changes in the composition of the atmosphere can disturb this balance. This will change the average temperatures on Earth, and consequently affect life on Earth (3, 103).

Measurements of Antarctic ice samples taken at different depths show an atmospheric increase in CO_2_ levels over the past 250 years with a strongly accelerated increase since the 1950’ties (104, 105) and, since CO_2_ is a GHG, resultant changes in temperatures can be expected. The public discussion around mitigating climate change and reducing the GHG concentrations in the atmosphere has focused narrowly on limiting the emission of GHGs from the use of fossil fuel, and on increasing the removal of GHGs from the atmosphere, e.g., by planting more trees and by carbon-capture and storage in human-made deposits. Terrestrial and aquatic environments are, in these discussions, viewed as isolated, passive sinks that can incorporate GHGs into vegetation and bind them into organic material in soil and lake- and seabed. Animals are seen as contributors to GHGs, e.g., cattle and other ruminants have been singled out, whereas other important sources of methane emission, e.g., antibiotics as shown by Bollinger et al. (58), have been omitted from the debate. It is important to recognise that data on CO_2_ emissions or “the carbon footprint,” which plays a prominent role in the debate, almost exclusively represents direct GHG emissions resulting from the human use of fossil fuels and that it only includes emissions from sources *believed* to be important, i.e., the emission data are selective estimates using statistical data on human activity—they are not based on actual measurements of emission resulting from human activity.

An example of an important carbon source that generally is omitted in the debate and in carbon models is the release of carbon from natural storage, e.g., soil or ocean. A common carbon-offset strategy is to plant new trees without recognising and incorporating the fact that old forests store twice as much carbon compared to young forests (106). This means that a proper carbon budget needs to consider both the carbon that is removed from air as well as the carbon that is released from soil if the new planting involves clearing areas with any existing vegetation (107–109).

In general, indirect emissions caused by chemical pollution, e.g., antimicrobials, are not considered, despite a multitude of studies demonstrating the critical role of the Earth microbiome in controlling the biogeochemical cycles (2, 31, 70, 107, 108, 110–115). Considering that it was microbes that changed Earth’s atmosphere enabling our evolution and that studies confirm their continued importance in maintaining a balanced atmosphere, excluding them from these calculations is likely to result in misleading conclusions.

### Impact of antimicrobials

#### Calculating the impact of antimicrobials on soil carbon storage

**Figure 4:**
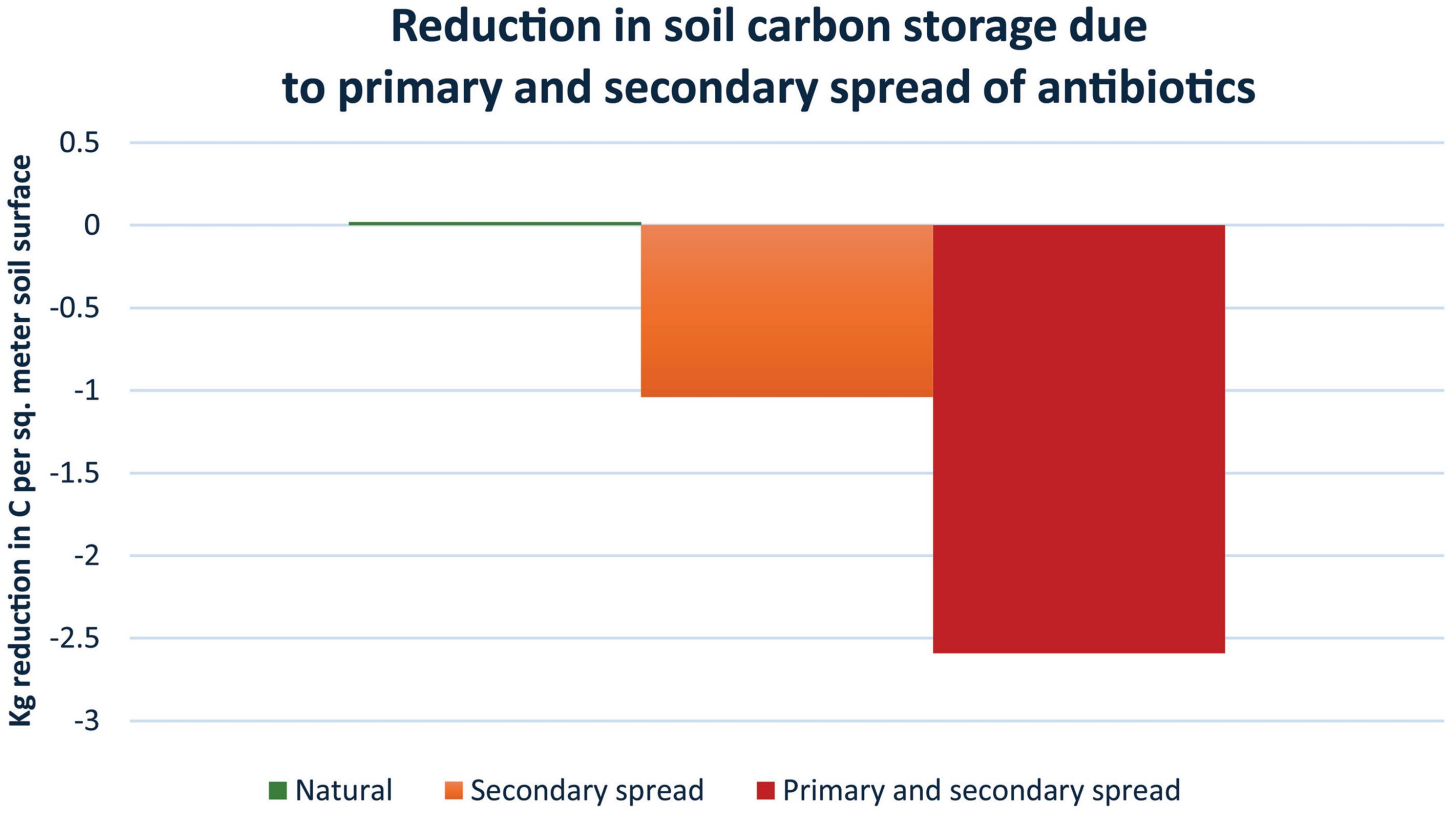
Reductions in soil carbon storage due to primary and secondary spread of antibiotics. Based on Roy et al. (20), the loss of carbon storage per sq. meter fertile soil surface is estimated in soil with native grazers (1.04 kg/m^2^), i.e., secondary spread of antibiotics as the animals are not receiving and therefore not directly contributing antibiotics to the soil (orange); and in soil with livestock grazers (2.59 kg/m^2^), i.e., primary spread as the animals are regularly receiving antibiotics as well as secondary spread from precipitation etc. (red) (see text for details). The loss is not an annual loss, but a general reduction in storage capacity with the released amounts of carbon ending up in the atmosphere, keeping in mind that the Earth and its atmosphere form a closed system. The reduction in storage capacity owing to antibiotics is considerable. For example, using these data it can be estimated that a standard course of tetracycline would lead to a reduction in carbon storage of 2.68 tonnes carbon or 9.84 tonnes CO_2_ (Supplementary material S1). This is equivalent to a standard car driving around the Earth 1.47 times.

Several studies have confirmed that antimicrobials result in the release of GHGs from soil and aquatic environments (19, 58, 65), but they did not allow the estimation of the long-term impacts of antimicrobials. A recent study by Roy et al. (20) made it possible to quantify the impact of antimicrobials on the ability of soil to store carbon (see S1 for details on the calculations). They monitored areas with livestock grazers, mainly cattle, who directly received antibiotics and spread this through their faeces (primary spread), and compared this to nearby areas with native grazers, who did not receive antibiotics (therefore causing no spread). Over a period of 10 years, the area with livestock grazers consistently had 1.55 kg/m^2^ *less* carbon stored in the soil compared to the nearby area with native grazers. Analysis of soil samples found, that the area with livestock grazers had 6.44 μg antibiotic (tetracycline equivalents) per kg soil or the equivalent of 1,932 μg antibiotic per m^2^ soil surface (300 kg soil per m^2^ soil surface). In comparison, the area with native grazers had a level of 2.59 μg antibiotic per kg soil or the equivalent of 777 μg antibiotic per m^2^ soil surface, which presumably originated as secondary spread from precipitation and predator led introductions. Owing to the extensive spread of antibiotics by precipitation followed by uptake by plants and animals with subsequent spread within the food nets, a true control area without antibiotic contamination no longer exists (116). Assuming a linear dose-relationship between the amount of antibiotics in the soil and the impact on carbon storage, it is possible to calculate how much extra carbon that could have been stored in soil if it had contained no antibiotics. The difference in concentration of antibiotic between livestock and native grazers per m^2^ is 1,932 μg minus 777 μg, i.e., 1,155 μg antibiotic per m^2^. This releases 1.55 kg carbon per m^2^, i.e., 1.34 g carbon per μg antibiotic or 1.34 kg carbon per mg antibiotic. Using this number (Figure 4), it can be determined that, in the area with livestock grazers, an extra 2.59 kg carbon could have been stored per m^2^ if no antibiotics had been present (1.34 g carbon per μg x 1,932 μg antibiotic per m^2^ soil surface); and in the area with native grazers an extra 1.04 kg carbon could have been stored per m^2^.

#### Determining the global impact

**Figure 5:**
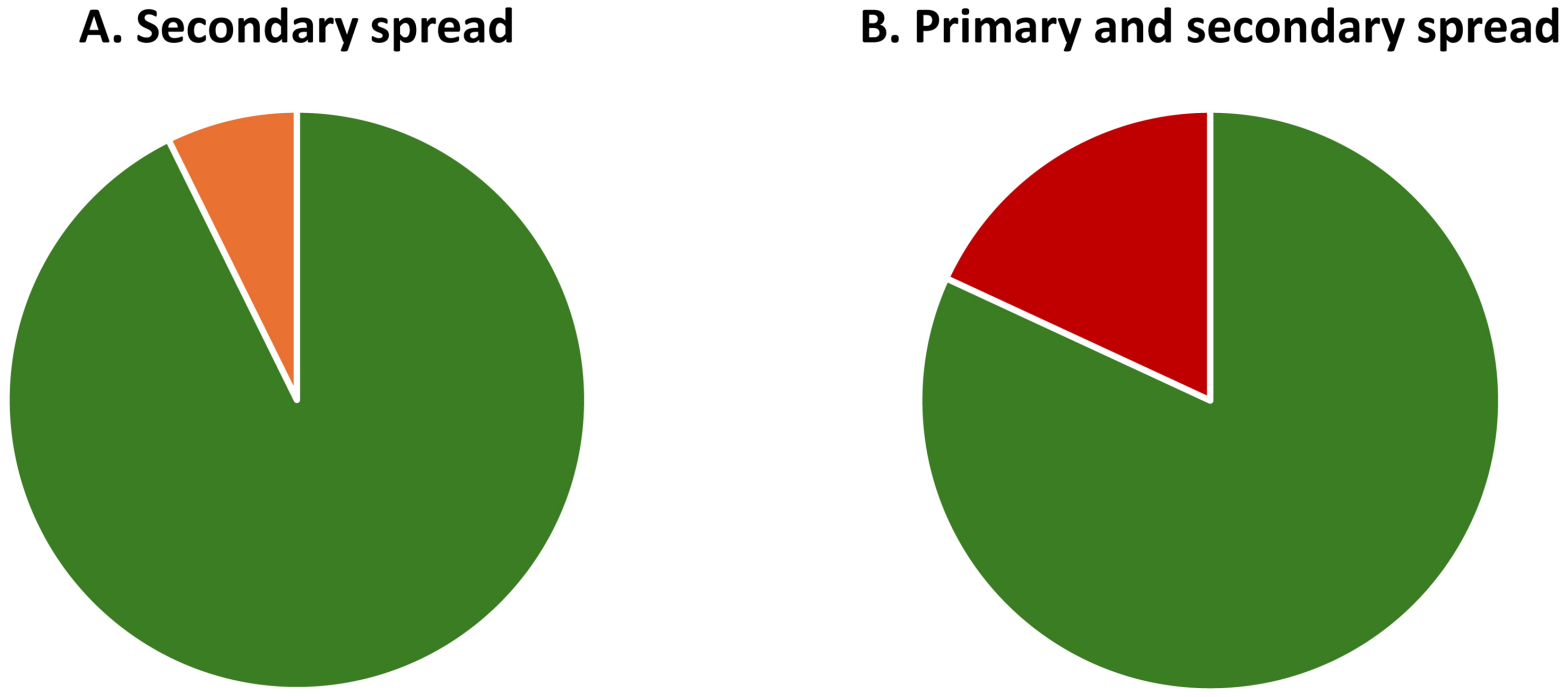
Potential storage capacity that can be realised by eliminating synthetic antimicrobials in fertile soil. Impact on capacity of primary and secondary spread of antibiotics. **(A)** Secondary spread in areas with native grazers: If the entire circle represents the amount of CO_2_ in the atmosphere in 2022, the orange section reflects the CO_2_ that could have been stored in soil if secondary spread of antibiotics had not occurred. The release corresponds to 194.8 billion tonnes CO_2_ or 7.3% of the total amount of CO_2_ in the atmosphere. **(B)** Primary and secondary spread in areas with livestock grazers: This shows the same as A, but for combined primary and secondary spread; this corresponds to 484.0 billion tonnes CO_2_ or 18.1% of the CO_2_ in the atmosphere.

To determine the global impact of antimicrobials, the amount present in areas with livestock grazers will not be representative of fertile land areas in general, because fertile land is put to many different uses and, consequently, not uniformly subjected to primary spread of synthetic antibiotics. Instead, data from areas with only secondary spread, i.e., with native grazers, will be more reliably relevant as secondary spread occurs everywhere. Bearing in mind that Earth and its atmosphere is a closed system, and assuming that all fertile land areas on Earth are comparable to the area with native grazers, i.e., with only secondary spread, it is possible to calculate how much of the carbon in the atmosphere that could have been stored in fertile soil if synthetic antibiotics had not been present in the soil (Figure 5). Fertile land constitutes about 10% of the Earth’s surface. Consequently, owing to the secondary spread of antibiotics, 53.1 billion tonnes carbon, which is the equivalent of 194.8 billion tonnes CO_2_, are no longer stored in soil compared to the condition with no synthetic antibiotics in the soil, but have instead been released into the atmosphere. The carbon no longer stored in the soil is the equivalent of 7.3% of the total amount of CO_2_ in the atmosphere. This is 5.2 times the calculated total CO_2_ emissions from human activity in 2022 (21). Furthermore, the contributions resulting from the lack of capture and storage of carbon due to antimicrobials from secondary spread in non-fertile land, vegetation above ground, and oceans are not included. Other studies similarly show that antimicrobials widely impact the storage of carbon in terrestrial and aquatic environments and lead to an increase in greenhouse gases (19, 58, 117), thereby providing support for the importance of these relationships.

#### Antimicrobials remain active and continue to exert their effects on the environment

**Figure 6:**
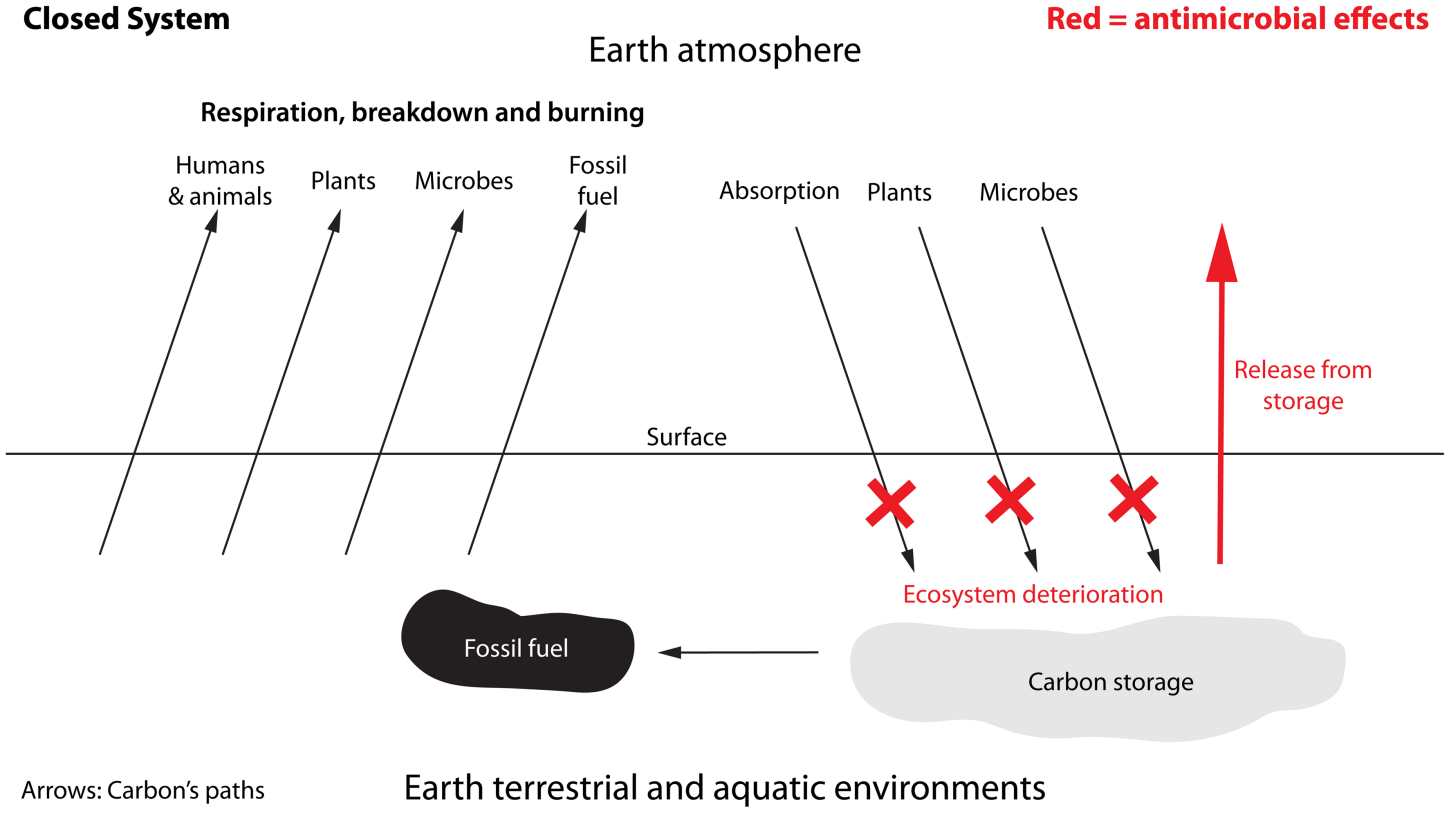
Sources contributing carbon to the atmosphere and carbon’s removal from the atmosphere. The diagram emphasises how antimicrobials do not directly contribute to the amounts of carbon being cycled, but instead impact the functioning of existing cycles thereby changing the amounts. In this respect antimicrobials can be seen as acting as a non-competitive inhibitor by not competing for access to carbon, but by disrupting the processes of the carbon-cycle. Arrows represent the movement of carbon through the carbon cycle. Unlike the burning of fossil fuel, which leads to the direct emission of carbon, the effects of antimicrobials will be indirect from affecting other cycles. Its impact on carbon may therefore not necessarily be an annual contribution but rather a reduction in the levels of carbon that can be absorbed and/or stored in soil.

The current climate debate primarily focuses on reducing or replacing the use of fossil fuels. Whilst this is a contributing factor, the burning of fossil fuel is the return of organic material that has been stored for a very long time, i.e., a process that the Earth and its systems have handled before. In contrast, antimicrobials will disrupt these foundational and interdependent systems that have evolved over an evolutionary timeframe and thereby fundamentally affect the ability of the Earth to maintain homeostasis (Figure 6). It can be argued that antimicrobial activity exists naturally in nature, but the half-life of these compounds is normally very short, and their impact will therefore be local, focused and short-lived. In contrast, synthetic antimicrobials are typically developed to be stable, enabling them to spread widely in the environment, e.g., 40–90% of antibiotics are excreted intact or as an active metabolite (65). Most eco-tox studies only measure the free fraction in water and use this to show how rapidly the substance is removed, but very many antimicrobials bind to substrates and remain active (66), meaning that most eco-toxicology reports underestimate the long-term role of these compounds.

The impact of antibiotics on the microbial environment has been confirmed (118, 119). Antiseptics, e.g., chlorhexidine, show extended environmental half-lives and contribute to resistance and cross resistance (8, 9, 120). Nano-silver is highly toxic to the microbes required for nitrogen cycling, a process essential for plant life (121–126). Surfactants, which reduce the surface tension of water, are antimicrobial. They also change the structure of soil, affecting the ability of plants to grow, they affect cloud formation and precipitation and, by interfering with the surface tension of the oceans, they reduce the absorption of GHGs from the atmosphere into the oceans (127–130). Other chemical groups such as PFAS (“forever chemicals”), pesticides, herbicides, preservatives, and food additives are known to have antimicrobial properties (14, 15, 108, 131, 132). Finally, newer studies show, that microplastics facilitate the transfer of antimicrobial resistance, which will exacerbate these effects (133, 134).

#### Climate change, fossil fuel, and shortcomings in modelling

An obvious question is, whether data are available to support the current assumption that human carbon emissions from the use of fossil fuels are the primary cause of changes in atmospheric CO_2_ concentration? Cyclic changes in atmospheric CO_2_-levels are common, but the current levels are substantially higher than in the past 800,000 years (105). The rapid increase began in the late 1950’ties and the timing correlates with increases both in the use of fossil fuel and in the use of antimicrobials (135, 136), meaning that both factors could be responsible. The next question is, whether there have been recent periods with reduced or increased human GHG emissions, *and* whether this impacted global CO_2_ levels accordingly? During the Covid pandemic in 2020, the burning of fossil fuels globally was very strongly reduced over a period of several months. The reduction in calculated CO_2_-emission levels was strong, but the measured global rise in atmospheric CO_2_-levels was unaffected (103, 137, 138). The lack of impact could possibly be attributed to global buffer-systems evening out these effects; however, from 1979 to 2004 and from 2004 to 2019, the rise in calculated emissions reduced and increased, respectively, relative to their projected path, without being reflected in the measured atmospheric level of CO_2_ either (138). In relation to a critical role of AMR, Oyelayo et al. (139) found higher regional temperature indicative of climate change in areas with high levels of AMR, suggesting detailed studies may be able to demonstrate a connection. Together, these observations suggest that the models used to calculate global GHG emissions focus too strongly on fossil fuel and are missing critical components, one of which could be antimicrobials causing CO_2_ to be released from the natural storages (sometimes referred to as sinks) and not captured into natural storages (19, 20, 65, 87, 117, 130, 140, 141). Ignoring any significant contributor to CO_2_-emissions will mean, that incorrect conclusions are reached and that inefficient strategies are developed and followed.

## The impact and consequences of using antimicrobials

### Impact of antimicrobials on the spread of AMR across species and habitats via mobile genetic elements (MGEs) and horizontal gene transfer (HGT)

When an antimicrobial is applied to a microbiome-hosting body surface of either a human or an animal, e.g., skin, gut (digestive tract), lungs (respiratory tract), or vagina, it reduces the number and percentage distribution of microbial species constituting the microbiota, i.e., microbial diversity and relative abundance, respectively, as well as causes AMR. Similarly, when an antimicrobial is applied to a surface in nature, e.g., soil, water, or foliage, or to a human-created surface in the city, be it buildings or pavement, like in individuals, it reduces the microbial diversity and supports the spread of AMR among the microbial inhabitants of that surface. AMR is linked to an increase in microbial virulence of the microbial strain on the host surface (47, 142, 143), which further curtails the diversity and strengthens the dominance of resistant, virulent microbes, thereby cementing the dysbiotic state of the microbiomes.

The fact that a species or strain is resistant to antimicrobials is not, in itself, a problem, provided that no antimicrobials are used on or around it. A consequential problem of AMR is, however, that resistance capabilities to the old antimicrobials do not disappear, develop extremely quickly to any new ones, and are linked to microbial virulence. AMR and virulence factors spread very effectively, across species and habitats (144). Unlike the mammal genome, the bacterial genome possesses a high degree of plasticity, i.e., it can very rapidly alter and diversify as a response to changes in the environment. It is dynamic and can gain (insert) and lose (exert) genetic information via many different types of mobile genetic elements (MGEs) such as plasmids, integrative and conjugative elements (ICEs), bacteriophages, gene transfer agents (GTAs) and many more (145).

MGEs are, in essence, functional, extrachromosomal gene sequences capable of replicating and of generating novel recombinant mobile elements with new gene combinations (146, 147). They are self-interested and semi-autonomous and can act both mutualistically or antagonistically to the host and influence many capabilities, including metabolism, motility, virulence, biofilm production, and AMR (148). Antimicrobial resistance gene sequences (ARGs) and many virulence factors are MGEs, frequently in the shape of plasmids (148, 149). MGEs all move fragments of DNA either within the host chromosome, or to other cells’ genomes, where they are either integrated into the host chromosome or remain as extrachromosomal entities contributing to the host genome. The MGEs, therefore, create innumerable possibilities of change and adaptation of the bacterial/microbial DNA. However, carrying MGEs can also reduce the organism’s competitive advantages, i.e., it can be associated with a fitness cost, depending on the environment and the specific circumstances (150, 151).

MGEs are quickly, easily and effectively shared with other bacteria across species, phyla and across habitats via horizontal gene transfer (HGT) (144). HGT does not require generational replication, i.e., the Darwinian traditional vertical transfer, and provides a considerably less time and energy consuming method of passing capabilities on to other cells, therefore facilitating a short response time when the bacteria are faced with an unknown or uncommon challenge. Because of the myriad of possibilities for recombination and insertion, these DNA segments often present genetic rearrangements assembled from many different taxa (152). It is also worth noting that the genome can change very differently in strains across the same bacterial species (153).

HGT can occur in three ways: (1) conjugation, which physically passes the gene sequence from the cytoplasm of the donor into the cytoplasm of the recipient. It is among others the transfer method used by the plasmids and ICEs; (2) transduction, which does not require such direct contact but can be visualised as small parcels of genetic material being picked up intracellularly, couriered to other cells and inserted into their cytoplasm. It is typically exemplified by bacteriophages and gene transfer agents (GTAs); and (3) transformation, which releases DNA from the cytoplasm into the extracellular environment either by secretion, programmed cell lysis or lysis following natural cell death, or as parcels in the shape of vesicles. In essence, transformation makes a free-floating pool of readily available DNA sequences, with a very diverse library of capabilities, freely available for other microbes, as well as eucaryotes (154), to actively pick up and use (155).

### MGEs are known for their role in the transmission of AMR

Apart from healthcare facilities, soil, aquatic environments, and wastewater treatment systems are major reservoirs of ARGs (149, 156–160). Free-floating ARGs, e.g., from dead bacteria or actively released vesicles, are able to persist in soil for at least 3 months (43) and for hundreds of years in deep-sea sediments (159), sea ice (161) and arctic glacial ice (162). They spread without the requirement of bacterial proximity, inter alia, via waterways and precipitation (43, 163).

With each use of an antimicrobial, be it antibiotics, antiseptics, disinfectants or others, e.g., in human or veterinary healthcare and food industry plants, the microbes develop new antimicrobial resistance gene sequences (ARGs). As bacteria have the capacity to accumulate ARGs, they not only can become resistant to multiple antimicrobials but the acquired ARGs will be used to assemble yet novel DNA sequences from ever more diverse origins (152). The pool of available gene sequences in the biosphere has been described as a single, common, shared resource for all bacteria to draw on (116). In other words, as the challenges to the microbes, e.g., the use of antimicrobials, are intensified, the tempo at which an ARG can establish a global presence has become nearly simultaneous (116), whereby it can be picked up practically everywhere across species (148) and phyla (154).

If the use of antimicrobials was reduced to an absolute minimum, it would reduce the prevalence of ARGs, but the resistance genes are unlikely to ever disappear. They would rather remain available in the global pool of extracellular DNA (159). However, data indicate that it is associated with a relatively high fitness cost to the microbes to carry these MGEs (150, 151, 164), and therefore, if the MGEs do not confer an advantage that is greater than the cost of carrying them, the carrying microbe will be at a disadvantage and over time either stop actively carrying them or be outcompeted. This means that we have the opportunity to reduce the frequency of MGEs conferring AMR gene material if we very considerably reduce our use of antimicrobials.

### The impact of antimicrobials on individual microbiomes

**Figure 7:**
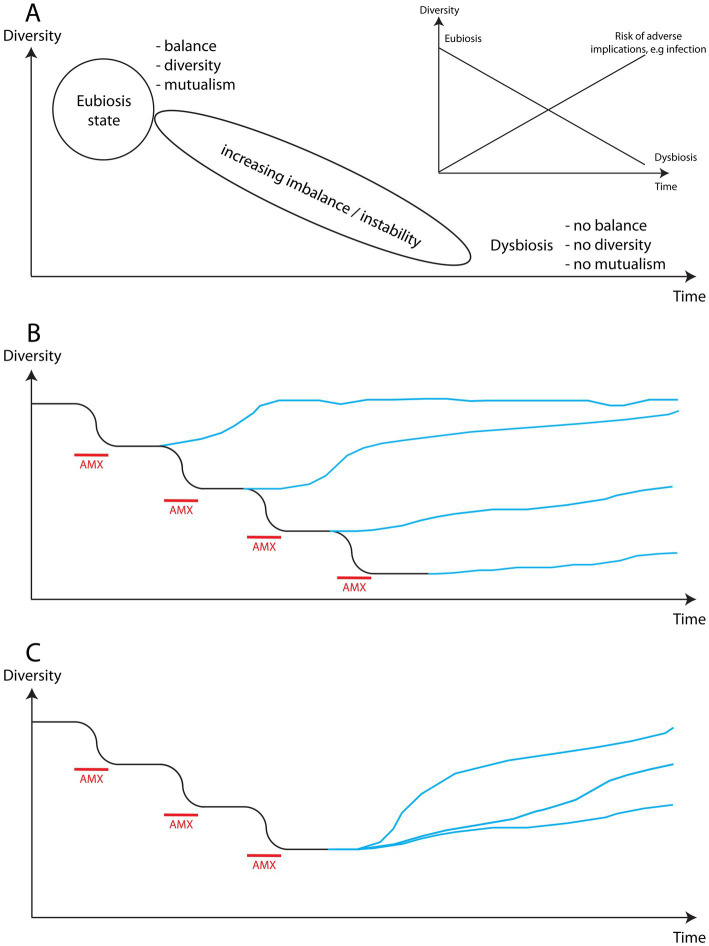
Schematic illustration of the impact of antimicrobial use (AMX) on the diversity of a microbiome. **(A)** Schematic representation of the path from eubiosis to dysbiosis of the microbiome characterised by increased instability. The insert highlights how the risk of disruption of the microbiome by external factors increases as it becomes increasingly unstable. **(B)** Each period of antimicrobial use increasingly reduces the diversity of the microbiome, making the return to a high level of diversity more difficult and time consuming until reaching a point where it may not be possible. **(C)** New studies show that it is possible to affect the level of diversity reached by the microbiome and its speed of recovery, suggesting that steps can actively be taken to support the recovery of affected environments.

A microbiome in a healthy state (eubiosis) is characterised by being in balance, diverse and with the inhabitants acting in mutualism, i.e., helping each other. Eubiosis is not a single, static, defined state, but rather a varying state with many possible fluctuations depending on circumstances, but with a stability characterised by diversity, mutualism and balance. A microbiome, or microbial ecosystem, is continuously affected and challenged by external factors that can severely affect its function, decreasing its stability to the point where it loses its ability to restabilise and reestablish an eubiotic state but instead reaches a state of dysbiosis, which is characterised by lack of balance, i.e., dominance by one or a few species, low diversity, and poor mutualism. This is shown in Figure 7A using diversity as the key parameter reflecting the state of the microbiome. The insert shows how the risk of complications increases as the microbiome becomes increasingly unstable and dysfunctional, e.g., increased risk of infection.

The use of an antimicrobial will reduce the diversity of a microbiome (Figure 7B). Each different type of antimicrobial will target a different sub-population within the microbiome thereby reducing diversity further, and each use of the same antimicrobial will exacerbate the lack of diversity already effected on the microbiome and obstruct any potential attempt of repopulation that could have been underway. Therefore, each exposure to an antimicrobial weakens the microbiome accumulatively destabilising the state of the microbiome. Each exposure will, consequently, reduce the ability of the microbiome to withstand attacks by pathogens or to prevent one or a few commensals from seizing control, and will thereby increase the risk of infection. For example, extremely severe, often fatal, antimicrobial-resistant infections such as necrotizing fasciitis (in skin microbiome) and *Clostridioides difficile* (in gut microbiome) are typically both rooted in dysbiosis characterised by very low diversity and strong dominance of an opportunistic or true pathogenic strain or species. In plants, exceptionally aggressive bacterial and fungal cankers, rot and wilt (165, 166) are examples of similarly aggressive infections allowed to develop owing to drastic reductions in diversity. Desertificated soil equally suffers from dysbiosis.

As a microbiome gradually slides from the balanced, diverse eubiotic state towards the imbalanced dysbiotic state, an increasing level of instability in the microbiome affects its functioning and impacts its capacity, e.g., long-term consequences were reported in humans even following normal antibiotic use (see Introduction). Studies have shown that plant diversity correlates with carbon storage (85, 167, 168) and that monoculture or few species cultures increase the prevalence of plant pest and illness (169, 170), highlighting that low species diversity in these ecosystems impact function in a manner similar to microbiomes. In town environments, air pollution impacts the diversity of the human respiratory microbiome (171) and can lead to increases in, e.g., child diabetes, asthma and allergies (172) and, similarly in plants, low air quality may increase the risk of disease, including foliar disease (165, 166). Given that the spread of antimicrobials and ARGs is both airborne and waterborne (30, 163, 173–175), it is impossible to compartmentalise spread when seeking illness prevention and new treatment approaches. Rather, this new understanding reflects the connectedness of all species and all environments and how similar the consequences are at different ecosystem levels, e.g., the consequences of low diversity in microbial and macro ecosystems.

Studies confirm that when the use of antimicrobials is stopped the systems will gradually move towards reestablishing eubiosis (6, 176–179). However, the new state of eubiosis will usually be different compared to the state prior to the exposure to antimicrobials (180), and the recovery will be complicated by the fact that once certain, often abundant, microbial species have fallen below a certain level, they are no longer able to survive and bounce back but instead disappear from that microbiome permanently (54).

The time and path to reach a new state of eubiosis will depend upon the type of microbiome and the degree of severity with which it was affected. An encouraging finding in both human and plant systems (Figure 7C) is that it is possible to positively impact the level of diversity in a recovering microbial environment. This means that we can influence the robustness of the recovery of these ecosystems (170, 177) and increase the level of carbon storage (85). However, any improvement would be counteracted by returning to the use of antimicrobials.

## RRR—replace, remove, reduce

The findings indicate that, to counteract climate change, one important goal is to reduce the total exposure of nature to synthetic antimicrobials. Over time, this should lead to a reduced prevalence of AMR and increased diversity and balance in the microbial environment with a resulting increased storage of carbon. The most effective approach is to *replace* the use of antimicrobials with a non-antimicrobial approach, because this circumvents the entire problem, assuming the new approach is clinically equal or superior and environmentally friendly. The second most effective approach is *removal*, e.g., to develop compounds that are rendered inactive and harmless as quickly as possible after having served their purpose, e.g., through metabolic breakdown, rapid environmental degradation (129, 181), or by destroying them before entering the environment, e.g., in community care collecting medical waste containing antimicrobials and disposing of it sensibly, as opposed to it currently being disposed of with normal household waste. Finally, *reducing* the amounts used, e.g., by not prescribing antibiotics for conditions against which they are known to be ineffective, and by using diagnostic tools to confirm that the specific bacterial strain is sensitive to the chosen antibiotic before prescribing them.

### Treatment of infection

Replacing antimicrobials by novel approaches will be the most effective solution and should be the aim. This, however, requires that novel treatments are identified, which is challenging (182, 183). Another more practical hindrance is the integration of new treatments into healthcare, which on average takes 17 years from an invention has been demonstrated effective and approved until it is used routinely, with about half of the inventions not surviving this delay (184). For example in wound care, current standard care is primarily based on antimicrobials even though they are known to be ineffective (185–187) and the US FDA consequently has characterised wounds not healing spontaneously as an unmet clinical need (188). The amount of antimicrobials used in wound care is substantial, e.g., in the UK alone, 559 tonnes of antibiotics will be used in 2025 in the treatment of wounds without providing any clinical benefits (189).

#### Treating wound infection via the microbiome-immune axis

A new technology, micropore particle technology (MPPT), has been available since 2016, which uses only physical forces to interact with the wound microbiome, i.e., no antimicrobial action. These actions disrupt the microbial defence systems, whereby the host immune system regains the ability to regulate the wound microbiome and restore eubiosis. Wound infection, including AMR infection, can now be removed, allowing wound closure to proceed, including in immunocompromised patients (189–194). The approach is also effective in treating highly aggressive infections such as necrotizing fasciitis as well as spreading infections that respond neither to amputations nor to aggressive regimes of antibiotics and antiseptics (195).

**Figure 8:**
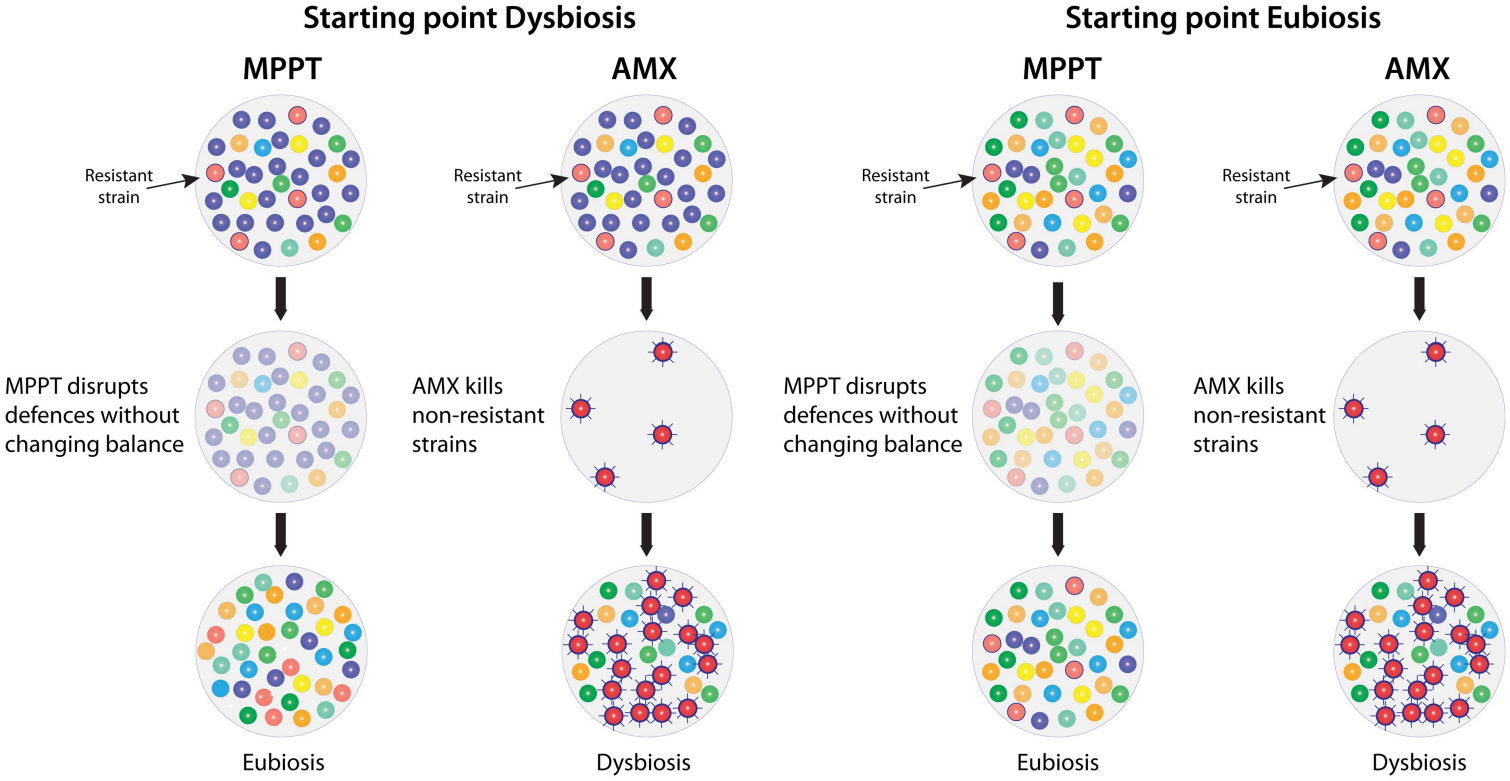
Comparison of the effects of MPPT vs. antimicrobials on the infected (left columns) and the healthy (right columns) microbiome of a wound with a resistant bacterial strain present. MPPT acts by disrupting bacterial defences (toxins and biofilm) without killing any organisms, whereas antimicrobials will kill the bacteria unless they are resistant to its effects (second row). MPPT will therefore not change the composition of the healthy microbiome and, in the infected microbiome, the immune system will selectively be able to remove the bacteria that need to be removed in order to reestablish eubiosis. In contrast, antimicrobials will kill all sensitive strains, whereas resistant strains will survive and often become hyper-virulent due to the exposure to the antimicrobial. The removal of sensitive strains will give the resistant strain a competitive advantage because it unhindered can populate the now bare area. AMX: Antimicrobial.

Unlike antimicrobial approaches, MPPT does not affect the diversity or the number of microbes in the microbiome, because it does not kill anything. Instead, it temporarily disarms all the microbes allowing the immune system to regain control. This has two implications. First, as shown in Figure 8, the microbial balance in areas without infection is not affected by the treatment, whereas the balance, when using antimicrobials, is severely disturbed with the risk of causing long-term effects as previously described. Second, MPPT exerts no external selection pressure on the microbes, because it does not kill anything and is consequently unlikely to lead to the development of resistance, i.e., it decouples the issue of resistance from MPPT. Essentially, any approach that places a general selection pressure on microbes will result in resistance, e.g., resistance to chlorine in drinking water and to UV-light have been found (196, 197). Bacterial communities actively respond to new dangers by developing new defence capabilities, where the individual members of the community each use a trial-and-error approach to identify an effective solution, and, once this has been identified, it is rapidly shared across the colony (45). Therefore, it is safe to assume that all antimicrobial approaches will result in resistance at some point.

Microbes also try to develop defences against the immune system but, unlike human-made treatments, the immune system co-evolves as the microbes change their tactics. In comparison, we first need to understand what happened, then we have to develop a new treatment, this needs regulatory approval, and finally it needs 17 years to come into routine use. Compared to microbes and the immune system, our responses are therefore very slow and ineffective, as clearly illustrated by our increasing inability to treat infections despite the danger of AMR having been known since 1917, when resistance to arsphenamine, the first widely used antibiotic introduced in 1910, was seen (135, 136, 198, 199).

The principle of supporting the bacterial-immune system collaboration is not unique to MPPT as studies have shown positive bacterial-immune system interactions in colorectal cancer (200) and in head and neck squamous cell carcinoma (201). These observations further point to a new important research field within the immune-bacterial axis, which, owing to MPPT, is known to be feasible and can be developed into new treatments. The challenge will of course be to identify feasible methods to support these collaborations. As such immune-microbial supporting research to *replace* antibiotics is likely to take time, the immediate, interim, short-term solutions are more likely to resort to *reducing* the environmental impact of antibiotics with a more traditional mode-of-action (202–204), but with a short half-life.

### Infection control and prevention in healthcare settings, agriculture and industrial plants

In the realm of infection control and prevention, e.g., in healthcare settings, the spread of life-threatening multi-resistant infectious diseases represent a considerable and recurrent problem (205–207). We have, for example, for many years endeavoured to keep our hospitals free from spreading severe infections by reducing the bioburden, i.e., the number of microbes, on surfaces. However, where this approach is used extensively, hospitals are plagued by highly resistant virulent bacterial strains, e.g., the high mortality rates on neonatal wards indicate that this strategy is unsuccessful (208).

In a clean-room, the combination of disinfectant cleaning, controlled entry, and highly filtered, contained air can reduce the bioburden, but in a space with ambient air and traffic of individuals, this is not possible. The bioburden cannot be reduced but will only be substituted by a different microbial population, which is what repeated use of antimicrobials in the shape of disinfectants do in healthcare and food industry settings. However, as they alter the microbiome in these spaces, they disrupt its stability and leaves weak, unstable patches and even temporarily free spaces for pathogens to populate and prosper. Also, whilst the disinfectants may kill the bacteria, they do not necessarily disrupt or remove their genetic material. This means that the MGEs, including ARGs and virulence factors, from the killed bacteria are left as a free-floating pool for the next patient’s bacteria/microbes to pick up, use and pass on.

From natural biological systems, it is known that surfaces are protected by their microbiome and that high diversity and mutualism is a requirement for stability and a healthy state (54, 209–211). The microbiota, i.e., the population of microbes pertaining to the microbiome, may fluctuate according to many environmental and internal factors, but the core microbiota is stable and a well-established biofilm protects the system. Contrary to common belief, biofilm generally plays a positive, protective role on surfaces, including for example on human skin (212). The microbiome therefore occupies the whole surface space leaving no free space for outsider microbes to populate and gain a foothold.

#### Using microbiomes to control and prevent the spread of infection

A replacement strategy to disinfectants could consequently be to keep surfaces hygienic without the use of antimicrobials and to allow natural diverse communities of microbes to settle and flourish in such a manner that *they* keep pathogens out. This is the strategy used by bodies, plants and ecosystems, and in light of the disastrous environments that we have created in our healthcare environments, it seems likely that working in harmony with our natural microbiomes instead of against them, would be a more productive and effective approach to controlling environments and preventing infections. This approach is not novel but may be what Florence Nightengale relied upon, when insisting on allowing fresh air into the wards (213, 214).

### An experiment in the development and impact of AMR on health—the International Space Station

An unintended long-term human experiment has further illustrated how the use of antimicrobials on a healthy microbiome is, in itself, disease-causing. On the International Space Station (ISS), the crew wash their skin and hair exclusively with a cocktail of antimicrobials agents, i.e., specially designed “non-rinse-soap.” The ISS is a closed system with only rare additions of new microbes, when supplies or new crew arrive. The state of the skin microbiome is therefore the direct result of the impact of all the stressors on the skin whilst in space. The result was a very high prevalence of antimicrobial resistant strains and the crew reporting impaired wound healing and changes in skin and hair structure, i.e., typical signs of dysbiosis (215, 216). Wang et al. (217) have shown that the skin microbiome is directly involved in wound healing and tissue regeneration and that this system is disrupted by antimicrobials. The process leading to such dysbiosis is illustrated in Figure 1, bottom row. On the ISS, there will be the added contributions of microgravity and radiation, but the use of antimicrobials will disrupt the ability of the skin to respond optimally to such stressors and mitigate their impact. Such a disruption will also impair other external bodily surfaces, e.g., lungs and gut, including their provision of essential factors to the body. Unless solved, this will also be a limitation on deep space exploration. The ISS and the Earth are both closed systems. The observations on the ISS are therefore likely to be mirrored on Earth, although at a different time scale due to their difference in size. The prospect, however, is that the Earth gradually will become uninhabitable to life-forms whose health depends on microbes.

## Discussion

The key conclusions that can be drawn are that human, animal, plant and environmental health are interdependent and that microbial health in the sense of diversity, balance, and mutualism is an equal necessity for all. When this is disrupted, everyone is affected in essentially the same manner—the specific symptoms may differ, but the biological basis is the same. We therefore need to update our view of microbes and realise that any use of antimicrobials invariably is associated with considerable collateral damage. However, we also know that, if we refrain from using antimicrobials, ARGs lose their importance and will decrease in prevalence over time.

Antimicrobials affect microbial health, and soil carbon storage has been shown to strongly depend on microbial health. Consequently, this means that GHG levels are impacted by our use of antimicrobials. However, the current climate debate focuses exclusively on GHG emissions caused by the burning of fossil fuels and it excludes contributions from other aspects of human activity, e.g., the use of chemicals that damage the Earth microbiome, i.e., the very system that created the existing atmosphere in the first place. Our destruction of the microbial environments affects the Earth at a very fundamental level and will disrupt the ability of the Earth to compensate for human activity (Figure 6). Whereas reduction in GHG emissions from fossil fuels is essential, the impact of emission reductions will be minimal if we continue disrupting the primary controllers of our atmosphere, namely the microbes. This is comparable to symptomatic versus curative treatment in healthcare. Whereas symptomatic treatment can help alleviate and even prolong the decline, it does not address the root cause and therefore, cannot restore the patient to health. A very large body of scientific evidence points to the importance of microbial systems in controlling and regulating Earth’s environment and, by not including these in the debate, we are most likely ignoring the root-cause of climate change. Changing the focus from carbon emissions to environmental sustainability and ensuring that a broader range of relevant factors and their impact on Earth are included in the models, would allow us to weigh factors against each other to reach better solutions for both the planet and its inhabitants, including solutions that would be acceptable to most people and therefore easier/possible to effectively implement.

The overall finding is therefore that a large body of data indicate that the increase in AMR is not due to climate change but that increases in AMR strongly contribute to climate change, i.e., the reverse of what is normally assumed. Given the urgency of addressing climate change, steps to limit the release of antimicrobials into nature should, therefore, be taken immediately, i.e., there is no need to wait for new research because the spread of AMR is an established problem, and it is well-known that all chemicals with antimicrobial effect contribute to AMR. The impact on Earth of antimicrobials is a function of their level of use and their use should therefore, as a first step, be replaced where possible and be restricted to areas where they are genuinely and demonstrably needed. There is currently an extensive use in a long line of non-essential areas, e.g., clothing, cosmetics, paints, plastics, limescale removers, and all-purpose cleaners with no critical, justifiable need, and policies to eliminate this use should be implemented immediately and without transition periods (218) because all of these products were until recently readily produced and consumed without the need of antimicrobials. Also, carbon and climate models should be changed to incorporate both direct and indirect impact of activities on GHGs, for example the use of antimicrobials or the clearcutting of our old forests (109), and to include all the most relevant factors, including capture, storage *and* release of carbon, to allow direct comparisons of the way different activities impact the climate. The healthcare sector is responsible for an extensive use of antimicrobials, and, whereas this is justified, the use should be strongly limited to areas where equal or better alternatives are not yet available *and* where they are shown to offer clear clinical benefits.
