# Supplementary material for: The contribution of antimicrobials and antimicrobial resistance to climate change and a possible way to reverse it whilst still offering high quality healthcare—a conceptual analysis
Source: Front Public Health. 2025 Jul 15;13:1644086. doi: 10.3389/fpubh.2025.1644086 (PMC12303994; doi:10.3389/fpubh.2025.1644086)
Supplement: Supplementary file 1 [file Data_Sheet_1.PDF]

Supplement 1 Calculations

Table S1. Amount of tetracycline released into nature following a single course

Dose	Daily	Days	Total	Breakdown/sewage plant	**Released**
0.5g	2g	10	20g	90%	**2.0 g**

Estimation of amount of tetracycline that escapes metabolic breakdown and is not caught in the sewage treatment plant. Tetracycline is excreted essentially unchanged via the kidneys and environmental break-down has been found to result in active metabolites.

Table S2. Weight of 1 square meter soil

	**Assuming:**	**Total weight**
1 cubic meter weighs 1.3 to 1.7 tonnes	1.5 tonnes	1,500 kg
1 sq. meter at 20 cm depth		**300 kg**

Calculation of the average weight of 1 sq. meter soil surface at a depth of 20 cm.

Table S3. Tetracycline concentration in soil with native grazers vs. livestock grazers and impact on Carbon

	**Tetracycline per kg soil and per square meter soil**		**Difference between Livestock and native**		**Native**	
	**μg/kg soil**	**μg/m² soil**	**Carbon kg/m²**	**CO_2 kg/m²**	**Carbon kg/m²**	**CO_2 kg/m²**
**Native**	2.59	777			1.04	3.82
**Livestock**	6.44	1,932				
**Difference**	**3.85**	**1,155**	1.55	5.68		

One sq. meter soil weighs 300 kg. The atomic mass of carbon is 12 and oxygen 16, meaning that 12 kg of Carbon lead to 2*16+12 kg CO_2=44kg CO_2 Thereby, 1 kg carbon equals 44/12 kg CO_2= 3.67 kg CO_2. A loss of 1.55 kg/m^2 C therefore becomes 1.55 x 3.67 = 5.68 kg/m^2 CO_2.

Table S4. Effect of 1 course of tetracycline or of the treatment of 1 pressure ulcer on CO_2 storage in soil

**1 course of tetracycline**	Average 2g released	**9,841 kg CO_2**<br>**9.84 tonnes CO_2**
**1 pressure ulcer**	1.46 doses /ulcer x 2g	**14,368.25 kg CO_2**<br>**14.37 tonnes CO_2**

Table S1 found that 1 course of tetracycline led to the release of 2 g of tetracycline into the environment. Roy et al. (2023) found that 1,155 μg tetracycline in soil results in 1.55 kg less carbon or 5.68 less kg CO_2 being stored per m^2 soil surface (Table S3). Therefore, dividing 2,000 mg tetracycline by 1.155 mg tetracycline per m^2 soil surface equals 1,732 m^2. This is multiplied by 1.55 kg carbon per m^2 soil surface, equalling 2,684 kg C per course tetracycline, or multiplied by 5.68 kg CO_2 per m^2 soil surface, equalling 9,835 kg CO_2 per course tetracycline. This therefore shows how much *less* carbon or *less* CO_2, respectively that is bound in soil owing to one course of tetracycline. Guest et al. (2018) found that new pressure ulcers on average are treated by 1.46 prescriptions of antibiotics, allowing the calculation of treatment impact on carbon in soil.

Table S5. Less Carbon or CO_2 bound in fertile land areas due to antibiotics

**Fertile Soil Surface (10%)**		**T Loss/km²**	**Billion T Loss over fertile land**
**50,960,000 km²**	C-loss	1042.727	53.1
	CO_2 loss	3823.333	194.8

Fertile areas make up about 10% of the Earth surface. Multiplying this area of fertile soil surface in sq. meters by the loss of C (1.04 kg/m^2) or CO_2 (3.82 kg/m^2) per sq. meter the total loss can be determined. This only includes fertile land areas, i.e. non-fertile land areas, oceans, and lakes are not included in the calculations.
